# Inappropriate Asystole Detection in Early Postoperative Phase after Loop Recorder Implantation

**DOI:** 10.5402/2011/146062

**Published:** 2011-04-17

**Authors:** Miriam Bortnik, Eraldo Occhetta, Andrea Magnani, Anna Degiovanni, Paolo Marino

**Affiliations:** Cardiology Division, Azienda Ospedaliera Maggiore della Carità, 28100 Novara, Italy

## Abstract

The implantable loop recorder is a useful diagnostic tool for patients with unexplained syncope. The capability to automatically detect and store arrhythmic events, implemented in the last generations of these devices, can further improve the diagnostic yield, but this feature can be compromised by inappropriate detection of false arrhythmias. We herein report the case of a patient in which several inappropriate activations of long-lasting asystole occurred in the two days following the implant, probably because of an intermittently loose contact between the device and subcutaneous tissue for a small pocket haematoma.

## 1. Introduction

The implantable loop recorder (ILR) is considered nowadays a powerful tool for the investigation of unexplained syncope and for transient events that suggest cardiac arrhythmias [1, 2]. The first generation of these devices (Medtronic Reveal 9525, Minneapolis, Minn, USA) was only capable of making patient-initiated recording by means of an external, hand-held device (Patient Activator); the following generations (Reveal Plus 9526, Reveal DX 9528 and Reveal XT 9529) have also the capability to automatically detect and record arrhythmic events. The role of new automatic detection algorithms in improving the diagnostic utility of ILRs is still not well established [3]. We herein report the case of a patient in which 19 episodes of inappropriate detection of long-lasting asystole were recorded in the two days following ILR implantation; we hypothesize that this phenomenon was related to transitory signal loss because of imperfect device contact with the subcutaneous tissue probably due to small pocket haematoma.

## 2. Case Report

**Figure 1:**
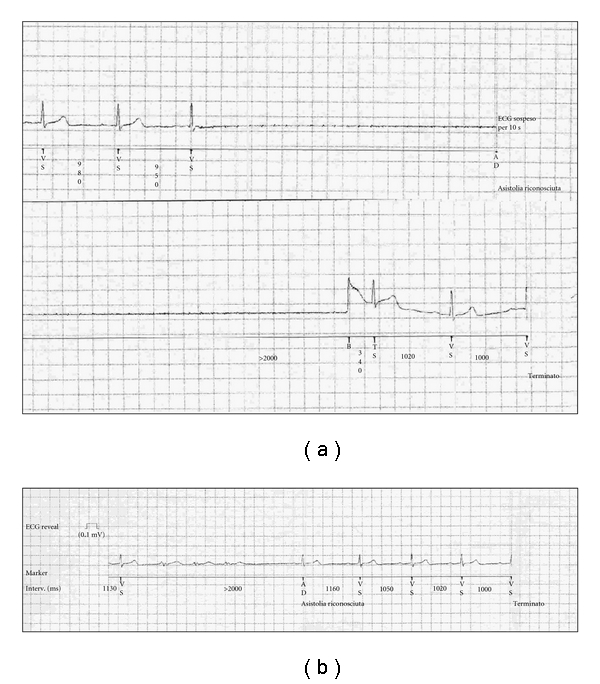
(a) and (b) inappropriate auto-detection of asystole. (a) shows an example of inappropriate long-lasting (42 seconds) asystolic pause detection from signal loss artefact recorded soon after ILR implant. (b) shows another example of inappropriate detection of a briefer pause related to undersensing of ECG signal amplitude later detected.

**Figure 2:**
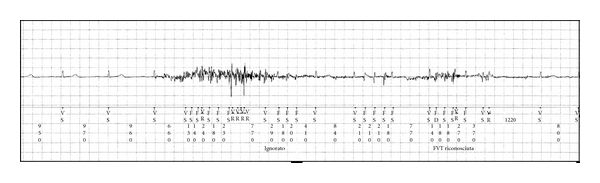
Inappropriate auto-activation of fast ventricular tachycardia due to oversensing from signal noise artefact.

A 74-year-old female patient was admitted to our Cardiology Department because of several episodes of dizziness and syncope. She had a history of hypertension and was receiving ACE-inhibitor. ECG on admission showed sinus bradycardia with a phase of junctional rhythm. An extensive cardiological investigation which included echocardiography, 24 hours Holter monitoring, Tilt test, and invasive electrophysiological study could not establish an aetiology. An ILR (Reveal DX model 9528) was implanted. The device was inserted into subcutaneous tissue of the left pectoral region; intra-operative real-time electrocardiographic telemetry showed reliable R wave sensing. The patient was discharged from hospital the following day; the device pocket appeared to be in good conditions. At the scheduled followup, three months after ILR implant, the patient was asymptomatic with no clinical events reported; at telemetry interrogation, 30 episodes of auto-activation, all inappropriate, have been stored. In 19 episodes, long phases of false asystole detection (up to 140 seconds of maximal duration) were related to signal loss artefact (Figure 1(a)). It is noteworthy that all these episodes of inappropriate prolonged asystolic pauses detection had been recorded during the first two days after the device implant and no more recorded subsequently. In 2 cases, inappropriate activation, which was recorded one month after ILR implant, was related to false asystole detection for brief undersensing of ECG signal amplitude (Figure 1(b)). In the remaining 9 events, recorded beginning from three days after the implant, inappropriate activation was due to false fast ventricular tachycardia detection related to noises (Figure 2). We have hypothesized that, in our patient, prolonged inappropriate autodetections of asystole in the early postoperative period were related to transitory signal loss because of suboptimal device contact with the subcutaneous tissue, probably due to a small swelling for a minimal pocket haematoma which rapidly subsided preventing further inappropriate detections with these characteristics. On the contrary, the following inappropriate autoactivations for undersensing of R wave or oversensing of noise signal artefact recorded in our patient represent a quite common phenomenon which, in our case, occurred despite the implemented new sensing and detection scheme. 

## 3. Discussion

The ILR is considered a valuable tool in patients with recurrent unexplained syncope following a negative baseline workup. The more recently developed versions of this device include an autoactivation function to supplement patient activation. It has been designed to capture asymptomatic arrhythmic events or symptomatic events missed by manual activation. Unfortunately, the ILRs may be subject to interference from commonly encountered electronic devices, such as antitheft surveillance systems and magnetic resonance imaging cameras [4, 5]; significant telemetry interferences have been observed also with cellular telephone [6] and more recently with a media player [7]. ILR correct functioning could be also hampered by other artefacts causing the false detection of arrhythmias, device memory saturation, and overwriting of appropriately detected episodes; these include sudden decrease in R wave amplitude during normal sinus rhythm and arrhythmias, undersensing by transient loss of ECG signal because of device amplifier saturation and oversensing related to T wave and myopotentials [8]. A previous study of Ng et al. [3], reported a very high incidence of inappropriate auto-activations (83%) in 50 consecutive patients implanted with Reveal Plus 9526. In the last ILR generation, automatic detection algorithms have been significantly improved and Brignole et al. [9] have recently demonstrated a decrease of inappropriate detections with the use of new sensing and detection algorithms with only a small reduction in the detection of appropriate episodes. Nevertheless, in our patient, who had been implanted with a latest version of the ILR, all the 30 autodetection events were inappropriate with a subsequent risk or relevant appropriate autoactivation episodes being erased. In our case, the majority of inappropriate autodetections has been recorded within two days since ILR implant and were related to signal loss probably because of a intermittently loose contact between the device and the subcutaneous tissue. This is a rather common phenomenon and is the reason why intrathoracic fluids accumulation monitoring (Optivol Fluis Status Monitoring) integrated in some implantable biventricular defibrillators manufactured by Medtronic is automatically initialized about a month after the implant [10]. The high prevalence of inappropriate auto-detection seems to limit the precocious reliability of this function in our patient; an eventual symptom-rhythm correlation using standard patient activation could be probably more useful, at least in this subject. Besides, probably in patients implanted with ILRs, an additional early device interrogation one week after the implant could be useful to recognize this type of troubleshooting and to avoid device consequent memory saturation.

## References

[1] Brignole M, Alboni P, Benditt DG (2004). Task Force on Syncope, European Society of Cardiology. Guidelines on management (diagnosis and treatment) of syncope-Update 2004. *Europace*.

[2] Giada F, Gulizia M, Francese M (2007). Recurrent unexplained palpitation (RUP) study-comparison of implantable loop recorder versus conventional diagnostic strategy. *Journal of the American College of Cardiology*.

[3] Ng E, Stafford PJ, Ng GA (2004). Arrhythmia detection by patients and auto-activation in implantable loop recorders. *Journal of Interventional Cardiac Electrophysiology*.

[4] De Cock CC, Spruijt HJ, Van Campen LMC, Plu AW, Visser CA (2000). Electromagnetic interference of an implantable loop recorder by commonly encountered electronic devices. *PACE—Pacing and Clinical Electrophysiology*.

[5] Gimbel JR, Zarghami J, Machado C, Wilkoff BL (2005). Safe scanning, but frequent artifacts mimicking bradycardia and tachycardia during magnetic resonance imaging (RMI) in patients with an implantable loop recorder (ILR). *Annals of Noninvasive Electrocardiology*.

[6] Trigano A, Blandeau O, Levy S (2005). Interference by cellular telephone with an implantable loop recorder. *Journal of Interventional Cardiac Electrophysiology*.

[7] Thaker JP, Patel MB, Shah AJ, Liepa VV, Jongnarangsin K, Thakur RK (2009). A media player causes clinically significant telemetry interference with implantable loop recorders. *Journal of Interventional Cardiac Electrophysiology*.

[8] Iglesias JF, Graf D, Pascale P, Pruvot E (2009). The implantable loop recorder: a critical review. *Kardiovaskuläre Medizinv*.

[9] Brignole M, Bellardine Black CL, Thomsen PEB (2008). Improved arrhythmia detection in implantable loop recorders. *Journal of Cardiovascular Electrophysiology*.

[10] Yu CM, Wang LI, Chau E (2005). Intrathoracic impedance monitoring in patients with heart failure: correlation with fluid status and feasibility of early warning preceding hospitalization. *Circulation*.

